# Oral supplementation of curcumin-encapsulated chitosan nanoconjugates as an innovative strategy for mitigating nickel-mediated hepatorenal toxicity in rats

**DOI:** 10.1007/s00210-025-03799-4

**Published:** 2025-01-21

**Authors:** Eman I. Hassanen, Neven H. Hassan, Sally Mehanna, Ahmed M. Hussien, Marwa A. Ibrahim, Faten F. Mohammed, Khaled Y. Farroh

**Affiliations:** 1 Department of Pathology, Faculty of Veterinary Medicine, Cairo University, P.O. Box 12211, Giza, Egypt; 2 Department of Physiology, Faculty of Veterinary Medicine, Cairo University, Giza, 12211 Egypt; 3 Department of Biotechnology, Faculty of Nanotechnology for Postgraduate Studies, Cairo University, Cairo, Egypt; 4 Department of Toxicology and Forensic Medicine, Faculty of Veterinary Medicine, Cairo University, Giza, 12211 Egypt; 5 Department of Biochemistry, Faculty of Veterinary Medicine, Cairo University, Giza, 12211 Egypt; 6 Department of Pathology, College of Veterinary Medicine, King Faisal University, 31982 Hofuf, Al Ahsa Saudi Arabia; 7 Nanotechnology and Advanced Materials Central Lab., Agricultural Research Center, Giza, Egypt; 8 Regional Center for Food and Feed, Agricultural Research Center, Giza, Egypt

**Keywords:** Curcumin, Gene expression, Nanoparticles, Nickel, Oxidative stress

## Abstract

Nickel pollution adversely affects human health and causes various disorders, mainly hepatic and renal dysfunction. The present work focused on a comparative evaluation of the pure form of curcumin (CU) with curcumin-encapsulated chitosan nanoconjugates (CS/CU NCs), on mitigation of the delirious effects of Ni on hepatorenal tissue. Forty-two male rats were allocated into 6 groups (n = 7 for each) as follows: (1) control, (2) CU, (3) CS/CU NCs, (4) Ni, (5) Ni + CU, (6) Ni + CS/CU NCs. After 30 days, blood and tissue (liver and kidneys) were collected to measure hepatorenal biomarkers, oxidant/antioxidant balance, inflammatory gene expression, liver and kidney histopathology, and immunohistochemistry. Results revealed disruption of hepatorenal functions, oxidative stress, and inflammatory markers at biochemical and molecular levels associated with severe hepatorenal histopathological alterations and abnormal immunohistochemical tissue expression for caspase-3 and cyclooxygenase-2. On the contrary, the treatment of Ni-intoxicated rats with CS/CU NCs markedly mitigated the adverse effect of Ni on hepatorenal tissue via regulation of oxidative stress, inflammatory, and apoptotic markers. The present study provides a novel nanoformulation for curcumin using CS NPs encapsulation that selectively targets the injured cells and improves the beneficial effect of CU via enhancing the antioxidant activity and regulating both inflammatory and apoptotic markers.

## Introduction

Modern industries have increased the number of hazardous metals by spreading them from the immobilized ore minerals, that people and animals are exposed to continuously. Hazardous metals can negatively impact any biological activity and affect several body systems based on the doses, duration, and routes of exposure. Nickel (Ni), a heavy metal, deserves special attention among the many environmental pollutants (Song et al. 2017; WHO 2021). People have repeatedly been exposed to nickel via ingestion of contaminated food and water or inhalation especially those who worked in mining, refining, electroplating, nickel powder metallurgy, nickel alloys, batteries, and nickel waste disposal (Chen et al. 2017; Lavinia et al. 2018). Among the three main organs where nickel accumulates in the body are the bone, kidney, and liver because the primary excretion methods for nickel are the bile and urine (Chen et al. 2017). Additionally, nickel induced structural and functional changes in other tissues including, skin, heart, spleen, testes, and GIT (Genchi et al. 2020).

Curcumin (CU), [1,7-bis(4-hydroxy-3-methoxyphenyl)−1,6 heptadiene-3,5-dione (diferuloylmethane)], is made by solvent extraction from turmeric or powdered rhizomes of Curcuma longa L (Naik et al. 2011). Many studies revealed the ability of CU to mitigate metal toxicity via its strong antioxidant, anti-inflammatory, and metal-chelating effects besides its non-toxic and non-mutagenic nature (Agarwal et al. 2010; Jakubczyk et al. 2020; Peng et al. 2021). It also demonstrates an extensive spectrum of biological activities, like free-radical scavenging, anti-inflammatory, antitumorigenic, antithrombotic, anti-spermicidal, antidiabetic, antimicrobial, antifibrotic, antivenom, antiallergic, and hypotensive properties (Shende and Samundare 2020; Alonso-Español et al. 2023; Yang et al. 2023; Rapti et al. 2024; You et al. 2024). Phospholipase, lipoxygenase, cyclooxygenase, leukotrienes, thromboxane, prostaglandins, nitric oxide, collagenase, elastases, hyaluronidase, monocyte chemoattractant protein-1, interferon-inducible protein, tumor necrosis factor, and interleukin-12 are just a few of the diverse molecules attributed the anti-inflammatory potential of curcumin in various laboratory studies (Liczbiński et al. 2020; Patel et al. 2020).

However, the curcumin’s benefits are frequently limited by several factors that affect its practical applications. For example, insufficient penetration and targeting efficacy, inadequate bioactive absorption, quick metabolization, lower pharmacokinetics and bioavailability, responsiveness to alkaline environments, heat, and light, lack of solubility in water, and physicochemical instability (Flora et al. 2013). Additionally, an extremely high dose of curcumin (CU) is needed to produce its therapeutic effect after oral supplementation, and it is considered another factor that restricts its practical application in medicine (Hussain et al. 2022). Curcumin's biological activity can be increased by incorporating it into nanocarriers via various techniques (Yallapu et al. 2015). This can increase the curcumin's bioavailability and solubility, as well as its long-term circulation and retention in the body, and it may also help to overcome physiological barriers and reduce the amount of CU needed to produce its benefits (Das et al. 2010; Li et al. 2013; Bhatia et al. 2016; Fonseca-Santos et al. 2016).

Chitosan was prepared by N-deacetylation of chitin, a natural source, and has a high safety profile (Hassanen et al. 2019a). Chitosan nanoparticles (CS NPs) have a variety of biological activities including antimicrobial, antioxidant, metal chelating, and cytoprotective effects (Hassanen et al. 2019a, 2021, 2022a; Hassanen and Ragab 2021; Mo et al. 2022). It is also used as an additive in animals’ food to improve their growth performance and immune status (Hassanen et al. 2023b). Recently, CS NPs have been used as carriers for various drugs and natural compounds to control and target their release (Hassanen et al. 2023a; AbdElrazek et al. 2023, 2024).

Despite extensive uses of curcumin, its detoxifying potential against nickel has not been studied elsewhere. Therefore, the current study is designed to compare the protective effect of CU and CS/CU NCs against Ni-induced hepatotoxicity in rats. Here, CS NPs encapsulation was used to improve the biological activities of CU after oral administration even at a very low dose instead of the use of ordinary curcumin powder. Additionally, a comprehensive insight into the mechanistic way of both CU formulations was provided via measuring either gene or immune expression of some inflammatory and apoptotic markers including nuclear factor kappa B, tumor necrosis factor-α, interleukin- 1β, caspase-3, and cyclooxygenase-2.

## Materials and methods

### Chemicals

Chitosan (molecular weight 50,000–190,000 Da, degree of deacetylation 75–85% and viscosity: 20–300 cP), acetic acid solution (1% v/v, ≥ 99.7%), Sodium tripolyphosphate (TPP) solution (0.06% w/v, technical grade, 85%), Tween 80, and curcumin (99%) were obtained from Sigma-Aldrich, USA. Nickel Sulphate hexahydrate 97% (NiSO_4_.6H_2_O), CAS NO. [10101–97-0] was purchased from CHEMBIO Co., PVT. Ltd., Navi-Mumbai, India.

### Preparation of curcumin-encapsulated chitosan nanoconjugates

The ionotropic gelation method was used to prepare curcumin-encapsulated chitosan nanoconjugates (CS/CU NCs). The anionic groups of sodium tripolyphosphate (TPP) interact with the amino groups on chitosan in the case of curcumin-loaded chitosan nanoparticles (Duse et al. 2018). In brief, acetic acid solution (1% v/v) was used to dissolve chitosan (CS) to create an aqueous solution (0.2% w/v). TPP solution (0.06% w/v) was added dropwise to the CS solution with vigorous stirring for 30 min. Curcumin solution was prepared by dissolving curcumin in ethanol at a concentration of 0.04 mg L-1. Next, curcumin was added dropwise to the CS solution and sonicated for five minutes at 50% amplitude in an ice bath in the presence of Tween 80.

### Characterization of the prepared nanoparticles

Using the X-ray diffraction (XRD) method, the chemical structure of the produced CS/CU NCs was evaluated. Phase analysis of the CS/CU NCs solution involved freeze-drying and grinding CS/CU NCs solution into a powder for X-ray bombardment. The corresponding XRD pattern was recorded using the Cu K radiation tube (= 1.54 A ^) operating in scanning mode (X 'pert PRO, PAN analytical, Netherlands) at 40 kV and 30 mA. The standard ICCD library that came with the PDF4 program was used to interpret the obtained diffraction pattern. A High-Resolution Transmission Electron Microscope (HR-TEM) was used to image the actual morphology of the prepared NPs (Tecnai G2, FEI, Netherlands). A diluted CS/CU NCs solution was ultrasonically sonicated for five minutes to reduce particle aggregation. Three drops of the sonicated solution were placed on a carbon-coated copper grid using a micropipette, and the grid was then allowed to dry at room temperature. Zeta sizer (Malvern, ZS Nano, UK) was used to estimate the average particle size distribution using the Dynamic Light Scattering (DLS) technique.

### Experimental design

The study was approved by the Cairo University Institutional Animal Care and Use Committee (IACUC) (Ethical number: Vet CU 25122023866, Date: 25/12/2023). Forty-two male albino Wistar rats weighing 140–150 g were procured from the Animal House of the Department of Veterinary Hygiene and Management at Cairo University, Faculty of Veterinary Medicine, Egypt. Rats were housed in plastic cages with free access to commercial pelleted food and water, all rats were acclimatized for two weeks prior to induction of experimental study.

Rats were randomly divided into six groups (n = 7 for each). Group 1 was maintained as a control group and was given distilled water. Groups (2&3) were given 1.5 mg/kg bwt curcumin (CU) and curcumin-encapsulated chitosan nanoconjugates (CS/CU NCs), respectively. Group (4) received Nickel Sulphate at 20 mg/kg bwt (1/10 LD50) (Pari and Amudha 2011). Groups (5&6) received 20 mg/kg bwt Ni with 1.5 mg/kg bwt of either CU or CS/CU NCs, respectively. All treatments were given via oral gavage for 30 days. Daily monitoring and periodical weighing of rats were conducted.

### Sampling

At the end of the experimental period, rats were anesthetized with a single intramuscular dose of Xylazine and Ketamine. Blood samples were taken and centrifuged at 3400 rpm/5 min to separate serum samples that were kept at 21 °c until they were needed for biochemical analysis. Afterward, rats were euthanized by cervical dislocation, and samples from the liver and kidneys were collected. Parts of them were fixed in 10% neutral buffer formalin till used for pathological analysis, and others were kept at −80 °c till used for oxidative stress evaluations and molecular studies.

### Biochemical assay

Biochemical analyses (Alanine aminotransferase (ALT), aspartate aminotransaminase (AST), Alkaline phosphatase (ALP), total proteins (TP), albumin (ALB), blood urea nitrogen (BUN) and creatinine were assayed using kits purchased from spectrum-Germany.

### Oxidative stress evaluation

The collected frozen tissue samples were homogenized using a cold buffer. Afterward, tissue homogenates were used to determine the levels of malondialdehyde (MDA), reduced glutathione (GSH), and catalase activity according to the methods described in the manufacturer kits (Biodiagnostic Comp., Cairo, Egypt).

### RNA isolation and qRT-PCR for *NF-κB, IL-1β, *and *TNF-α* genes

Using the RNeasy RNA Extraction kit (Qiagen) and the manufacturer's instructions, total RNA was extracted. Using spectrophotometric optical density measurement (wavelength, 260 and 280 nm), the amount and quality of RNA were ascertained (Abdelrahman et al. 2022). Following the manufacturer's instructions, the Revert Aid RT Reverse Transcription kit (cat. no. K1691; Thermo Fisher Scientific, Inc.) was used to create cDNA. The *TNF-α, NF-κB,* and *IL-1β* gene expression rates were measured using the ABI 7500 real-time PCR apparatus (Applied Biosystems; Thermo Fisher Scientific, Inc.) and SYBR Master Mix (Takara Bio, Inc., Otsu, Japan), following the manufacturer's instructions (Morgan et al. 2021). The primer pairs that were employed were included in Table 1. As the endogenous control, the expression of ACTB was used to standardize the expression levels of all target genes (Hassanen et al. 2022b; Mohamed et al. 2024). The initial denaturation step, which lasted 30 s at 95 °C, was succeeded by a second 35-cycle cycle consisting of two steps: 15 s at 95 °C and 30 s at 60 °C. The third step, which was conducted after the PCR melting curve was created, was as follows: 60 °C for 60 s, 95 °C for 15 s, and 60 °C for 60 s. The f_0_ method was used for the calculation of the fold change.

**Table 1:** Primer sequence, product size, and accession number of the studied genes

Genes	Forward primer	Reverse primer	Product	Accession no
*NF-κB*	ACCTGGAGCAAGCCATTAGC	AGTTCCGGTTTACTCGGCAG	234 bp	NM_199267.2
*TNF-α*	ACACACGAGACGCTGAAGTA	GGAACAGTCTGGGAAGCTCT	235 bp	NM_012675.3
*IL-1β*	TTGAGTCTGCACAGTTCCCC	GTCCTGGGGAAGGCATTAGG	161 bp	NM_031512.2
*ACTB*	CCGCGAGTACAACCTTCTTG	CAGTTGGTGACAATGCCGTG	297 bp	NM_031144.3

*NF-κB* nuclear factor kappa B, *TNF-α* tumor necrosis factor alpha, *IL-1β* interleukin 1 beta, *ACTB* Beta actin housekeeping gene

### Histopathology

The traditional methods of Bancroft followed for processing the formalin-fixed samples**,** using graded alcohol, xylene, and paraffin wax to obtain blocks (Bancroft 2012). Afterwards, blocks were cut using microtome into 4.5 µm sections that stained with H&E to examine for any histological alterations using Olympus BX43 light microscope. An Olympus DP27 camera connected to CellSens dimension software was used to take pictures. Utilizing a semiquantitative ordinal scoring system, the extent of cellular degeneration, necrosis, hemorrhage, and inflammatory cells infiltration was evaluated within tissue in 7 random microscopic fields/Sect. (5 sections/group). Five points were assigned to each lesion as the following:- (1) less than 25% tissue damage, (2) between 25 and 50% tissue damage, and (3) between 50 and 75% tissue damage, (4) more than 75% tissue damage (Hassanen et al. 2019b).

### Immunohistochemistry

The localization of both caspase-3 and cyclooxygenase-2 (Cox-2) was visualized within hepatorenal tissue sections using primary antibodies (Abcam, Ltd) for both immune markers and IHC-kit’s reagents (Power Stain 1.0 Poly HRP DAB Kit; Sakura) following the manufacturer instructions.

### Hepatic and renal content of nickel

Microwave digestion system (ETHOS One; Milestone, Sorisole, Italy) was used to digest 0.5 g tissue samples that had been placed in vessels with concentrated HNO3 and 30% H2O2 over the night. After that, flame atomic absorption spectroscopy was used to determine the amount of nickel content.

### Statistical analysis

SPSS Inc., Chicago, IL, USA, version 16.0 was used for statistical analysis. Means ± SEM was used to express the data. One-way analysis of variance (ANOVA). was used to compare the means (ANOVA). Whereas LSD post hoc test was utilized to evaluate mean differences across multiple comparisons. *P* ≤ 0.05 was the threshold for statistical significance.

## Results

### Characterization of CS/CU NCs

**Fig. 1:**
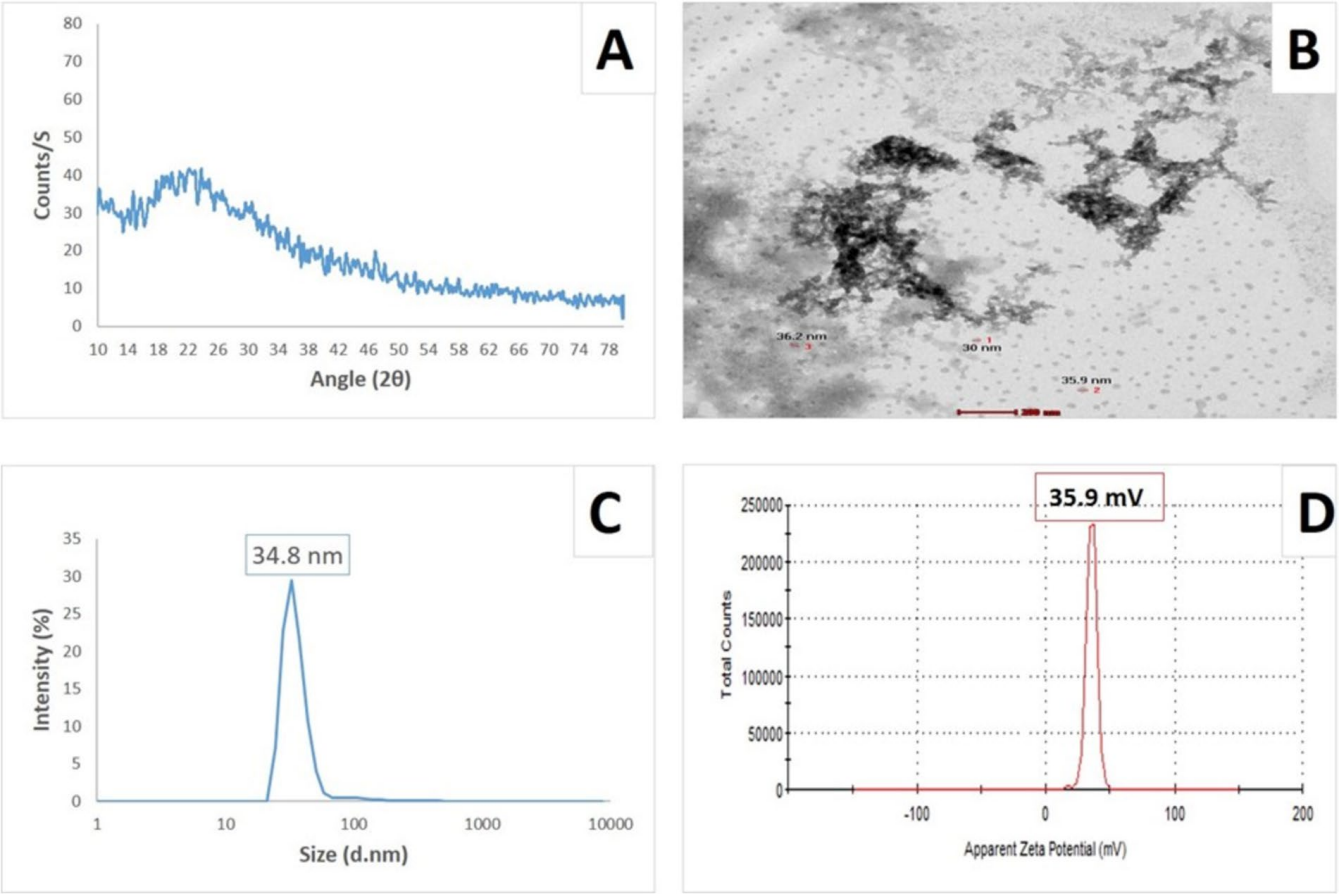
Characterization of CS/CU NCs. (**A**): XRD pattern analysis indicating the formation of CS/CU NCs. (**B**): HR-TEM image showing a nearly spherical shape of prepared CS/CU NCs with average size 34 nm. (**C**): Particle size distribution of prepared CS.CU NCs showing the average size of 34.8 nm. (**D**): zeta potential of prepared CS/CU NCs showing surface charge, zeta potential, + 35.9 mV

The XRD pattern of CS/CU NCs showed a broad typical hump peak (Fig. 1a). A typical HR-TEM micrograph of the CS/CU NCs was shown in Fig. 1b. The CS/CU NCs have a nearly spherical shape, smooth surface, and size range of about 34 nm. Figure 1c & d represented the DLS demonstrating a particle size 34.8 nm and zeta potential + 35.9 mV.

### Clinical signs and body weight gain

**Fig. 2:**
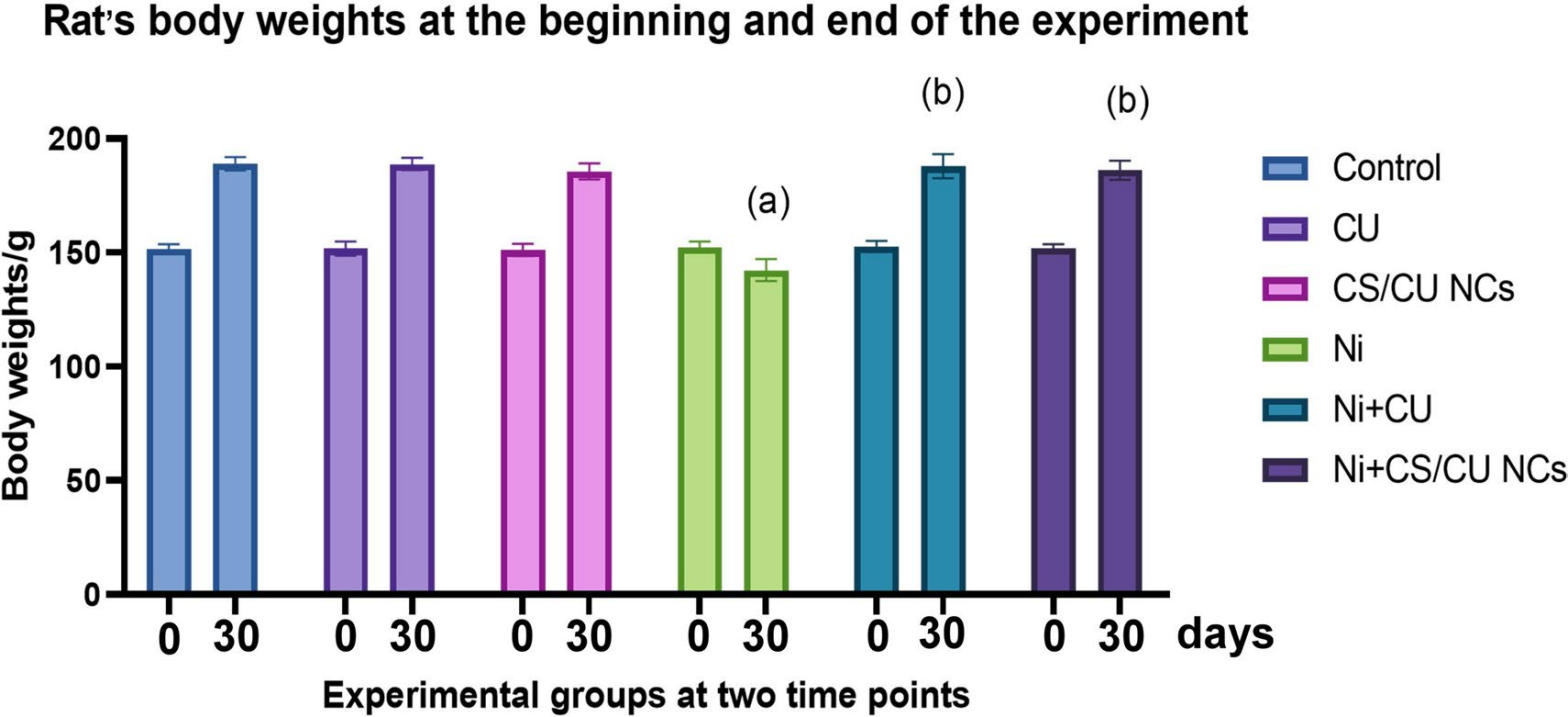
Rats body weights (Bwt) in different experimental groups at the beginning of the experiment (0 day) and the end (30 day). Data are expressed as Mean ± SEM (*n* = 7 rats/group), different superscript letters indicate significant differences at *P* ≤ 0.05

Throughout the experiment, rats in all groups showed no specific clinical features or mortality indicators. The body weight of Ni-intoxicated rats was markedly reduced compared with untreated control ones. In contrast, greater improvement in weight gain was achieved by nano-curcumin treatment compared to curcumin form (Fig. 2).

### Biochemical analysis

Data shown in Table 2 revealed a significant elevation of liver enzymes (ALT, AST & ALP) as well as kidney function parameters (BUN and creatinine) in the Ni group upon comparison with the control group. On the other hand, groups cotreated with Ni and either CU or CS/CU NCs showed a significant reduction of liver enzymes and kidney function parameters compared to Ni exposed group. However, the group cotreated with CS/CU NCs and Ni showed significant improvement when compared with the group cotreated with CU and Ni.

**Table 2:** Effect of Nickel sulfate, nano-curcumin, and curcumin on the biochemical parameters of various experimental groups

	Control	CU	CS/CU NCs	Ni	Ni + CU	Ni + CS/CU NCs
ALT (U/L)	61.15 ± 1.99^a^	64.17 ± 2.06^a^	62.17 ± 1.82^a^	93.13 ± 1.86^b^	76.33 ± 1.48^c^	66.83 ± 1.64^a^
AST (U/L)	40.48 ± 1.34^a^	37.93 ± 1.41^a^	38.88 ± 1.46^a^	62.00 ± 2.26^b^	52.83 ± 1.90^c^	42.17 ± 1.30^a^
ALP (U/L)	118.88 ± 3.22^a^	116.33 ± 2.51^a^	119.17 ± 2.57a	168.50 ± 3.35^b^	141.83 ± 2.68^c^	126.05 ± 2.92^a^
TP (g/dL)	6.66 ± 0.33^a^	6.25 ± 0.20^a^	6.72 ± 0.30^a^	5.75 ± 0.19^a^	6.48 ± 0.27^a^	6.40 ± 0.33^a^
ALB (g/dL)	3.65 ± 0.25^a^	3.75 ± 0.21^a^	3.37 ± 0.28a	3.67 ± 0.20^a^	3.53 ± 0.29^a^	3.53 ± 0.26^a^
BUN (mg/dL)	25.66 ± 1.76^a^	23.42 ± 1.49^a^	23.68 ± 2.11^a^	62.00 ± 3.51^b^	42.00 ± 3.62^c^	29.33 ± 2.17^a^
Creatinine (mg/dL)	0.59 ± 0.03^a^	0.62 ± 0.02^a^	0.55 ± 0.02^a^	1.29 ± 0.098^b^	0.82 ± 0.02^c^	0.60 ± 0.03^a^

Data are expressed as Mean ± SEM (*n* = 7 rats/group), values with different letters in the same row indicate that they are significantly different at *p* ≤ 0.05

### Oxidative stress evaluation

As shown in Table 3, Ni receiving rats exhibited significant elevation in MDA levels along with reductions in GSH and catalase activity compared with the control rats. On the other hand, cotreatment of Ni-exposed rats with either CU or CS/CU NCs significantly reduced the MDA levels and increased the activity of both antioxidants when compared to Ni receiving rats. However, the administration of CS/CU NCs significantly mitigated Ni-induced hepatorenal oxidative stress damage than CU.

**Table 3:** Effect of Nickel sulphate, nano-curcumin, and curcumin on the oxidative stress markers in liver and kidneys of various experimental groups

	Control	CU	CS/CU NCs	Ni	Ni + CU	Ni + CS/CU NCs
Hepatic tissue homogenates						
MDA(nmol/g)	2.7 ± 0.4^a^	2.8 ± 0.3^a^	2.6 ± 0.4^a^	7.8 ± 0.2^c^	4 ± 0.3^b^	3 ± 0.2^ab^
GSH (mg/g)	14.4 ± 2.1^a^	15.1 ± 1.1^a^	15.5 ± 1.9^a^	8.1 ± 0.8^c^	9.9 ± 0.7^b^	12.5 ± 0.7^a^
CAT (U/g)	1.4 ± 0.05^a^	1.1 ± 0.08^a^	1.5 ± 0.06^a^	0.3 ± 0.08^c^	0.7 ± 0.09^b^	1.2 ± 0.09^a^
Renal tissue homogenates						
MDA(nmol/g)	4.2 ± 0.5^a^	4 ± 0.8^a^	3.9 ± 0.4^a^	9.8 ± 0.9^c^	5.3 ± 0.4^b^	3.1 ± 0.3^a^
GSH (mg/g)	11.8 ± 0.8^a^	12.2 ± 0.5^a^	12.3 ± 0.7^a^	6.2 ± 0.4^c^	10 ± 0.5^b^	11 ± 0.7^ab^
CAT (U/g)	0.8 ± 0.07^a^	0.9 ± 0.05^a^	0.9 ± 0.06^a^	0.2 ± 0.05^c^	0.5 ± 0.03^b^	0.8 ± 0.05^a^

Data are expressed as Mean ± SEM (*n* = 7 rats/group), values with different letters in the same row indicate that they are significantly different at *p* ≤ 0.05

### RNA isolation and qRT-PCR for *NF-κB, IL-1β, *and *TNF-α* genes

**Fig. 3:**
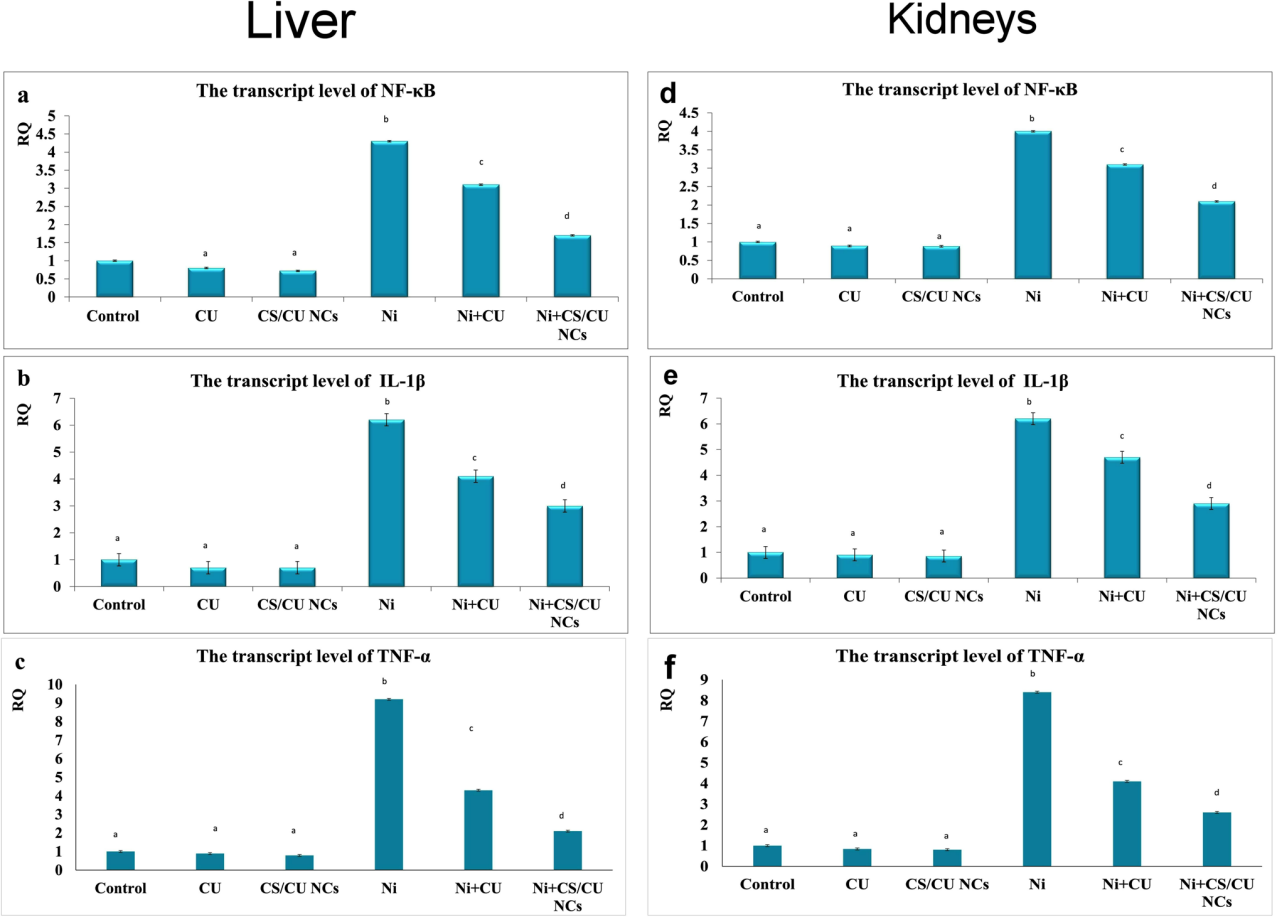
Quantitation of *NF-κB, IL-1β, TNF-α* mRNAs in both liver (**a**-**c**) and kidneys (**d**-**f**) of various experimental groups. Data are expressed as Mean ± SEM (*n* = 7 rats/group), different superscript letters indicate significant differences at *P* ≤ 0.05

In both the liver and kidney, the *NF-κB, IL-1β,* and *TNF-α* mRNA transcript amounts (Fig. 3) were significantly elevated in Ni-exposed rats. However, the curcumin and its nano form significantly alleviated the Ni effect by downregulation of the studied genes. The nano form of curcumin surpasses curcumin in its ameliorative effects.

### Histopathology

**Fig. 4:**
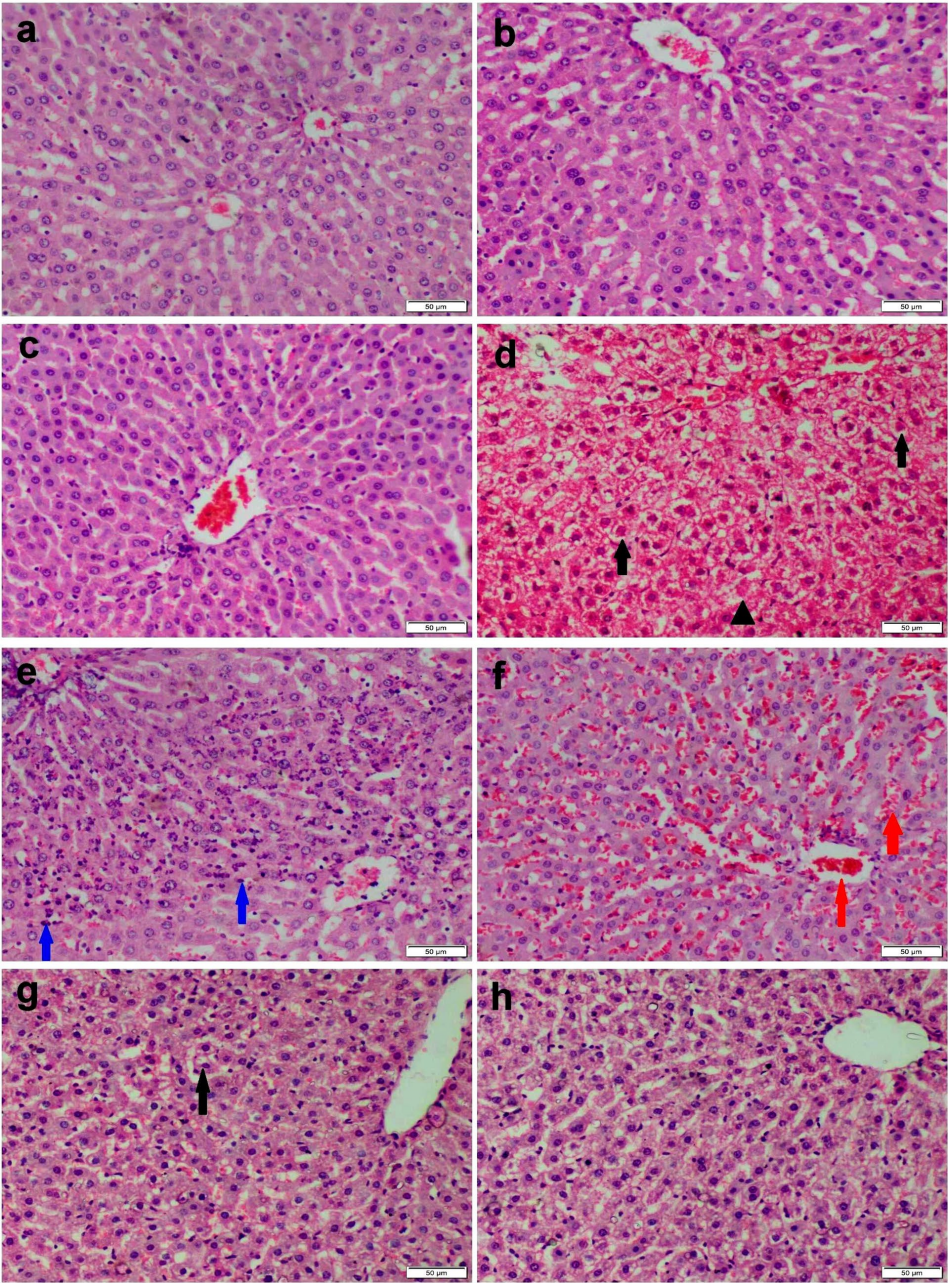
Microscopic appearance of H&E-stained liver sections obtained from various experimental groups. (**a**) control group, (**b**) CU receiving group, and (**c**) CS/CU NCs receiving group showed normal histological structure. (**d**-**f**) Ni receiving group showed diffuse vacuolar degeneration (black arrow), necrosis (triangle), congestion (red arrow), and inflammatory cells aggregation (blue arrow). (**g**) Ni + CU group showed moderate vacuolar degeneration (black arrow). (**h**) CS/CU NCs group showed normal histological architecture

The liver of the control group and those receiving curcumin either free or nanoconjugates showed normal histological patterns (Fig. 4a-c). Otherwise, the liver of Ni-exposed rats demonstrated extensive histopathological alterations. The most prominent lesions were severe diffuse hepatocellular vacuolation and necrosis (Fig. 4d). Some sections showed focal inflammatory cells infiltration and extensive sinusoidal dilation (Fig. 4e-f). On the other hand, the co-administration of CU either free or nanoconjugates with Ni improved the microscopic appearance of the liver to various degrees. CU-receiving group showed moderate hepatocellular vacuolation with single cell necrosis (Fig. 4g), while restoring of normal hepatic architecture was observed in nanoconjugates (Fig. 4h).

**Fig. 5:**
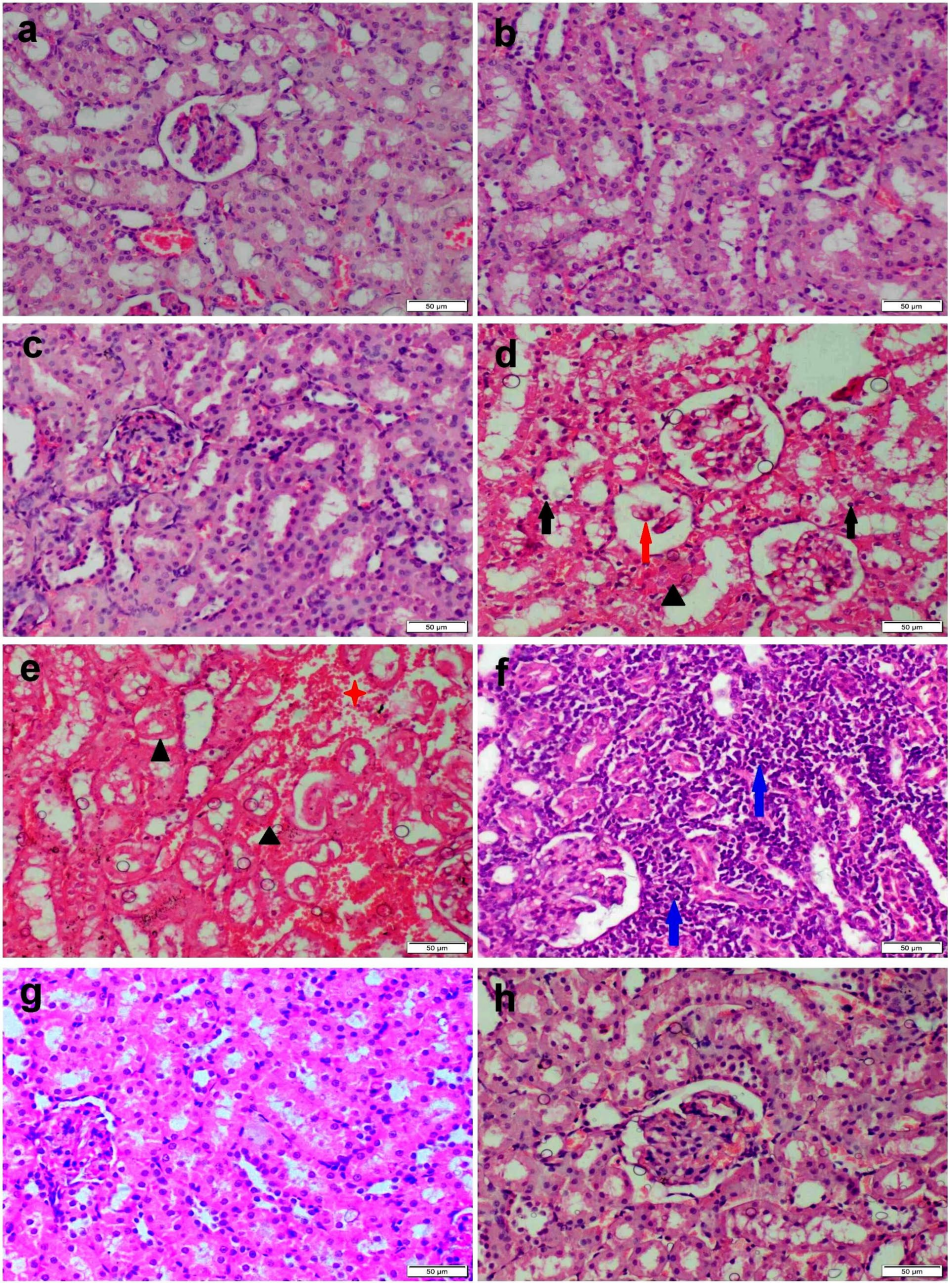
Microscopic appearance of H&E-stained kidney sections obtained from various experimental groups. (**a**) control group, (**b**) CU receiving group, and (**c**) CS/CU NCs receiving group showed normal histological structure. (**d**-**f**) Ni receiving group showed severe degeneration (black arrow) and necrosis of epithelial lining renal tubules (triangle), glomerular damage (red arrow), profuse interstitial inflammatory cells aggregation (blue arrow), and interstitial hemorrhage (red star). (**g**) Ni + CU group and (**h**) CS/CU NCs group showed normal histological architecture

The renal tissues of the control, curcumin, and nanoconjugate-treated groups showed normal histological structure (Fig. 5a-c). The Ni-intoxicated group showed severe necrobiotic changes involving the tubular epithelium associated with dilatation of peritubular blood capillaries and interstitial mononuclear cells infiltration (Fig. 5d-f). While the CU + Ni treated group showed moderate degeneration in the tubular epithelium (Fig. 5g). The treatment of Ni intoxicated group with CS/CU NCs succeeded in restoring the normal renal structure with no obvious histological alterations (Fig. 5h). The lesion score for different experimental groups was shown in Table 4.

**Table 4:** The grading score for histopathological alterations in liver and kidneys

	Control	CU	CS/CU NCs	Ni	Ni + CU	Ni + CS/CU NCs
Liver’s Histopathology score						
Degeneration/necrosis	0 ^a^	0 ^a^	0 ^a^	4 ^d^	3 ^c^	1 ^b^
Hemorrhage	0 ^a^	0 ^a^	0 ^a^	2 ^c^	1 ^b^	0 ^a^
Inflammatory cells	0 ^a^	0 ^a^	0 ^a^	3 ^d^	2 ^c^	1 ^b^
Kidney’s histopathology score						
Degeneration/necrosis	0 ^a^	0 ^a^	0 ^a^	4 ^d^	3 ^c^	1 ^b^
Hemorrhage	0 ^a^	0 ^a^	0 ^a^	2 ^c^	1 ^b^	0 ^a^
Inflammatory cells	0 ^a^	0 ^a^	0 ^a^	4 ^c^	1 ^b^	0 ^a^

Data are expressed as Median (*n* = 30 microscopic fields/group), values with different letters in the same row indicate that they are significantly different at *P* ≤ *0.05*

### Immunohistochemistry

**Fig. 6:**
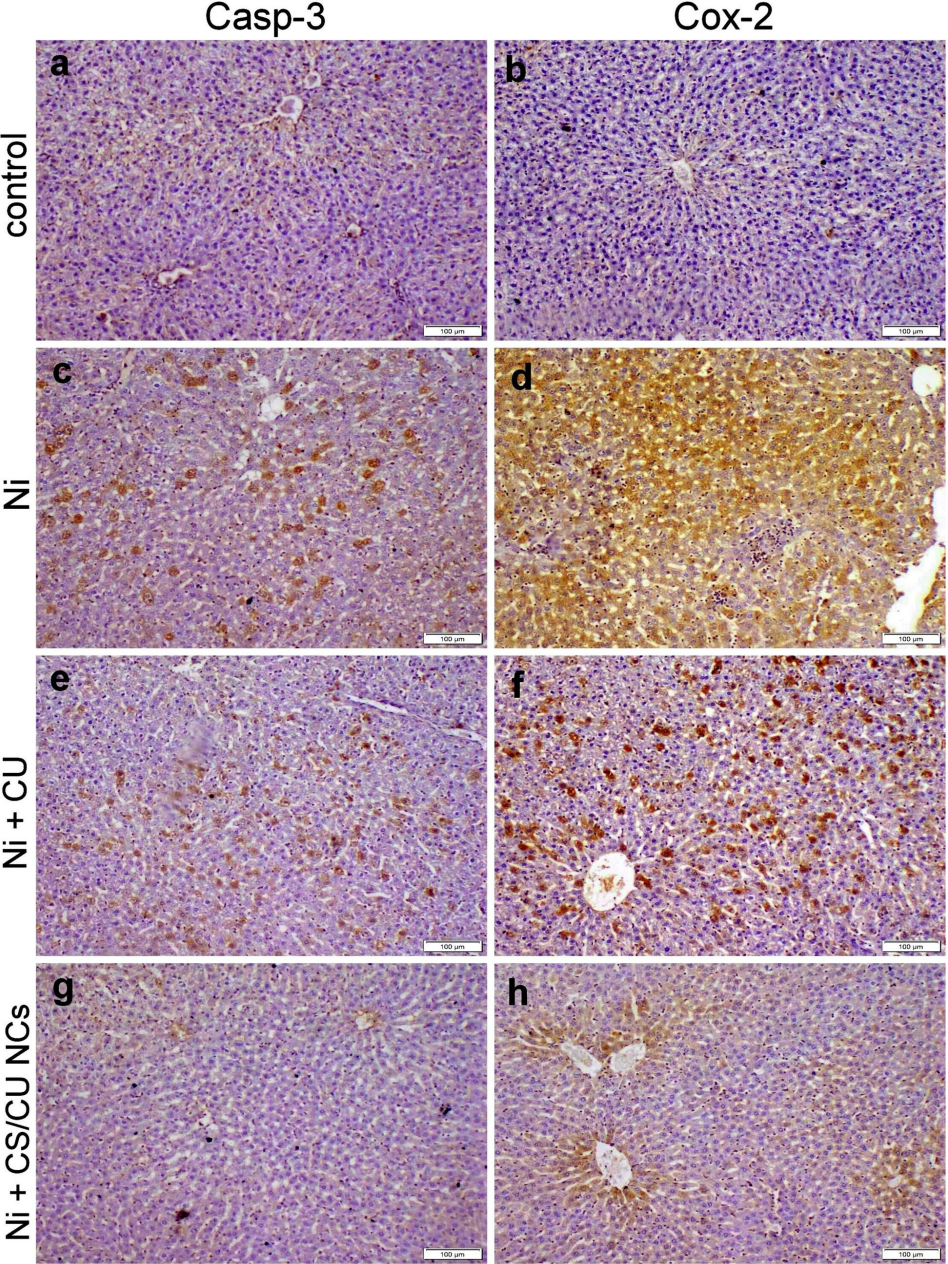
Caspase 3 and Cox-2 localization within the hepatic tissue of various treatment groups. (**a**, **b**) control group with negative casp-3 and cox-2 immunostaining. (**c**, **d**) Ni group displayed strong casp-3 and cox-2 immunostaining. (**e**, **f**) Ni + CU group exhibited moderate immunostaining reactions. (**g**, **h**) Ni + CS/CU NCs group displayed weak to negative immunoexpression

**Fig. 7:**
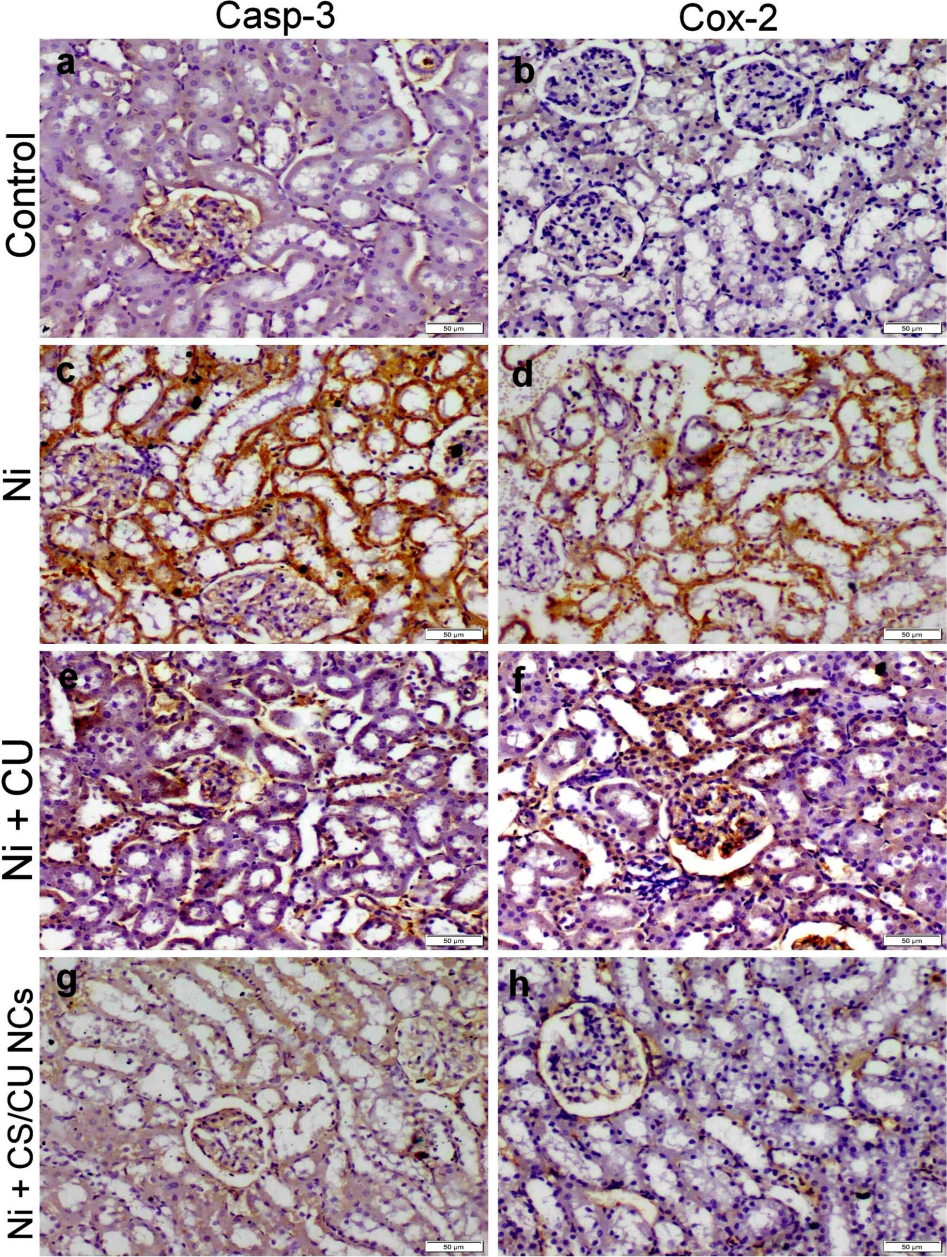
Caspase 3 and Cox-2 localization within the renal tissue of various treatment groups. (**a**, **b**) control group with negative casp-3 and cox-2 immunostaining. (**c**, **d**) Ni group displayed strong casp-3 and cox-2 immunostaining. (**e**, **f**) Ni + CU group exhibited moderate immunostaining reactions. (**g**, **h**) Ni + CS/CU NCs group displayed weak to negative immunoexpression

Figures 6 & 7 illustrated that Ni-receiving group exhibited strong caspase-3 and Cox-2 expression in hepatocytes and renal tubular epithelium compared with the control group. Moreover, the co-administration of free CU with Ni still showed strong casp-3 immunostaining with moderate Cox-2 immunopositivity in both hepatic and renal tissue sections in contrast to the Ni-receiving group. However, the administration of CS/CU NCs significantly reduced both caspase-3 and Cox-2 immunopositivity than CU.

### Hepatic and renal content of nickel

**Fig. 8:**
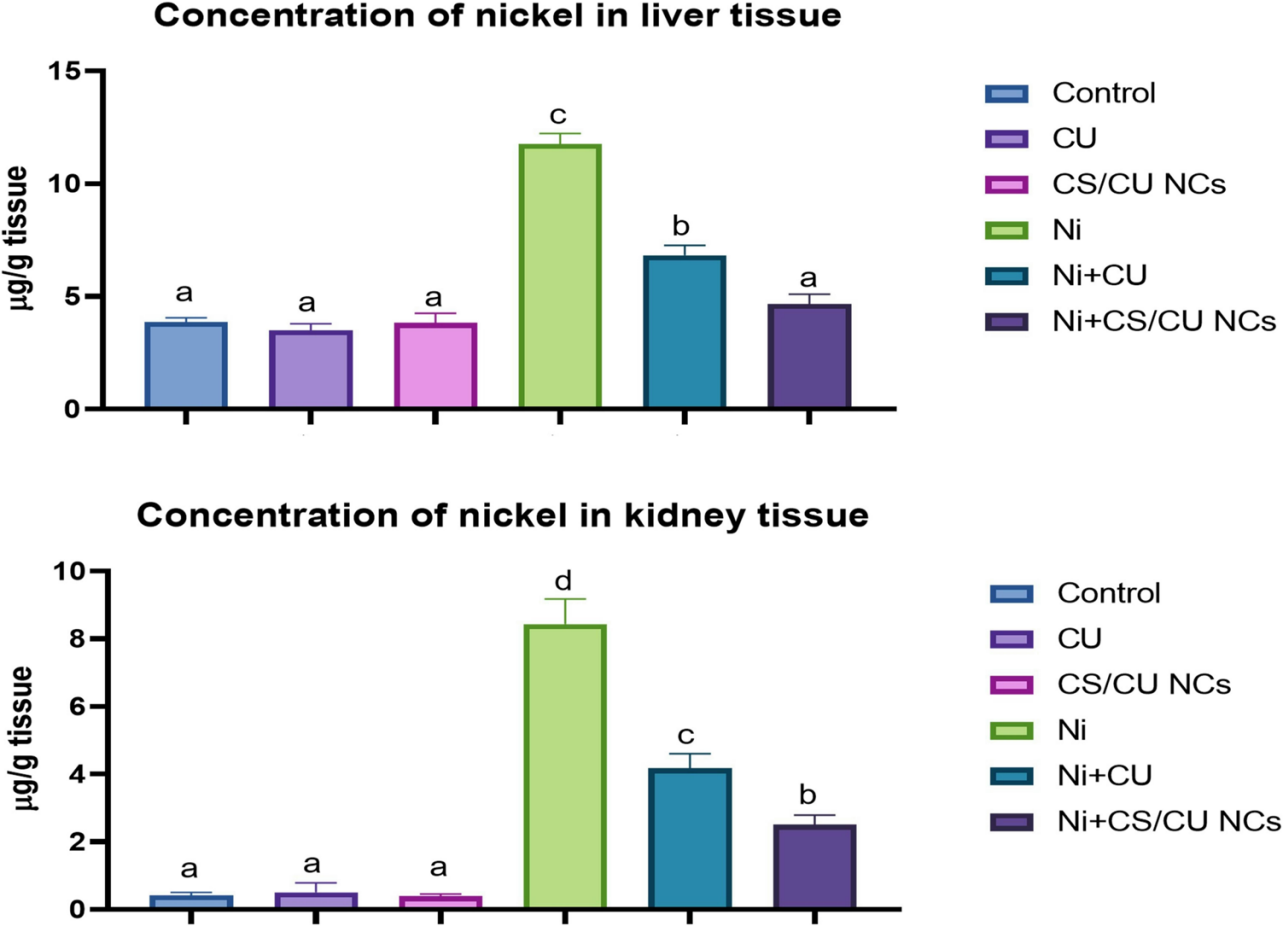
Concentration of nickel (Ni) in both liver and kidneys of various experimental groups. Data are expressed as Mean ± SEM (*n* = 7 rats/group), different superscript letters indicate significant difference at *P* ≤ 0.05

As shown in Fig. 8, Ni-receiving rats exhibited a significant elevation in hepatic and renal content of Ni across groups. On the other hand, the administration of either CU or CS/CU NCs with Ni significantly reduced the levels of Ni in both liver and kidneys in contrast to Ni-receiving rats. However, the level of Ni in both liver and kidneys obtained from CS/CU NCs-receiving rats was similar to those obtained from the control rats.

## Discussion

Recently, exposure to Ni become a health hazard problem and more efforts have been made to assess Ni toxicity and explore the molecular mechanisms that attributed to such toxicity (Song et al. 2017). The present work proved that Ni induced hepatorenal toxicity after thirty days of oral administration in rats. The key findings of hepatorenal toxicity included a significant reduction in body weight gain, disruption of organ biochemical functions, disruption of oxidative stress markers, and dysregulation of specific gene expressions that could justify the disruption of organ biochemical functions and develop hepatorenal pathology.

In line with our findings, several previous studies proved that the repeated oral administration of rodents with Ni could reduce the body weight gain and induce oxidative stress damage to liver and kidney tissues leading to organ damage and dysfunction (Sharma et al. 2024; Zhao et al. 2021). Oxidative stress-mediated lipid peroxidation caused membrane disruption and dysfunction leading to loss of enzyme activity that explained the elevated serum levels of ALT, AST, ALP, TP, ALB, BUN, and creatinine in Ni-intoxicated rats (Hassan et al. 2023). The disruption of organ biochemical functions induced by Ni toxicity was justified by the degenerative and necrotic pathological reactions that were detected in the liver and kidney. Several previous studies confirmed the deleterious effect of Ni on the liver (Naima and Zine 2012; Liu et al. 2017; Bozorgzadeh et al. 2023). Furthermore, the nephrotoxic potential of Ni was previously reported in rodents (Adeyemi and Elebiyo 2014; Bouhalit and Kechrid 2018; Guo et al. 2021).

In the present study, Ni induced elevation of oxidative stress parameters that resulted in activation of casp-3 and Cox-2 expressions in both liver and kidneys. Additionally, Ni provoked an inflammatory process in hepatorenal tissue as detected in histopathology and justified by upregulation of *NF-κB, IL-1β,* and *TNF-α* mRNA transcript amounts in Ni-exposed rats. The presented findings were justified by increased Ni levels in the hepatorenal tissue of Ni-intoxicated rats. Previous studies confirmed the ability of Ni to induce free radicles overgeneration, lipid peroxidation, and DNA damage (Akinwumi et al. 2020). Cempel and Janicka (2002) reported an increase in Ni levels in many organs such as the liver and kidneys of rats after oral daily intake. It is reported that the liver is the primary target for Ni aggregation due to the presence of metallothionein which highly binds with Ni (Amudha and Pari 2011)**.** It was reported that free radicles induced lipid peroxidation and mitochondrial membrane damage with the release of *cytochrome c* that initiated activation of the caspase cascade including casp-3 (Morgan et al. 2023). Caspase-3 activation is a crucial factor in the process of apoptosis mediated by toxicant-induced oxidative stress (Noshy et al. 2018). Cox-2 activation is a principal factor in the process of inflammation that is explained by its ability to convert arachidonic acid to prostaglandins which is responsible for the initial phase of inflammation including redness, swelling, and pain (Hassanen et al. 2024a, b). Consistent with the conclusions reached by Freitas and Fernandes (2011), our data indicated that Ni increased the secretion of proinflammatory cytokines by activating the NF-κB signaling pathway. NF-κB can trigger the transcription of proinflammatory cytokines, chemokines, and adhesion molecules and becomes extremely active at inflammation sites in various diseases (Liu et al. 2017).

Numerous attempts have been made to mitigate the negative impacts of nickel, including the application of natural antioxidants (Lavinia et al. 2018). Among natural antioxidants, curcumin (CU) has many beneficial effects including antioxidant, hepatoprotective, anti-inflammatory, and anti-apoptotic effects (Pandit et al. 2015; Jaroonwitchawan et al. 2017; Marslin et al. 2018). Although traditional turmeric or curcumin powder has been shown to have therapeutic benefits, researchers have noted certain drawbacks (Flora et al. 2013). Tremendous therapeutic potential of CU is hampered by its extremely low aqueous solubility, poor tissue absorption, and quick metabolism, highlighting the critical need for delivery vehicles to effectively distribute this amazing nutraceutical (Popat et al. 2014). A potential method to get around these limitations is to enhance the water solubility of CU by combining it with chitosan nanoparticles (CS NPs) (Popat et al. 2014).

The results of the current study clarified the ability of CS/CU NCs to reduce Ni-induced hepatic and renal injury more than the pure form of CU at the same dose. Recent study revealed that curcumin integrating with chitosan nanoparticles could increase water solubility of CU (Kumbhar et al. 2023). Das et al. (2010) reported that the preparation of CU in nanoformulation with chitosan and alginate by the ionic gelation method controlled their release to cancer cells**.** Akhtar et al. (2012) found that synthesized curcumin-coated chitosan nanoparticles increased the antimalarial activity in mice as well as improve metabolic stability and bioavailability. Previous research proved that polymeric nanoparticles loaded with curcumin showed excellent binding and cellular uptake, which increased their cytotoxic activity and inhibited the growth of tumors (Chaurasia et al. 2016). Solid lipid-loading curcumin nanoparticles have been demonstrated to increase its water solubility and decrease the activity of pro-inflammatory mediators induced by lipopolysaccharide, such as nitric oxide (NO), prostaglandins-E2 (PGE2), and interleukin (IL)−6, by blocking NF-κB activation (Nahar et al. 2015).

The present work revealed the potential role of CS/CU NCs in reducing Ni-induced oxidative stress-mediated hepatorenal pathology and downregulating several inflammatory and apoptotic markers including NF-κB, TNF-α, IL-1β, Cox-2, and Cap-3. Curcumin could modulate the pathways of arachidonic acid cascades (Cox-2 and lipoxygenase) in a variety of cell lines. Furthermore, it has been reported that curcumin inhibits the expression of the matrix metalloproteinase-3 and 13 (MMP-13, 3) genes in human chondrocytes by blocking NF-κB, and activating protein-1 (AP-1) and c-Jun-N-terminal kinase (JNK) pathways (Sohn et al. 2021)**.** It was demonstrated that CU NPs appear to be a suitable alternative to native curcumin in competing mastitis in mice (Suresh et al. 2018)**.** According to Hosseini et al., curcumin encapsulated in nano micelles exhibited greater anti-inflammatory activity than curcumin itself in preventing the development of lung injury induced by paraquat (PQ) (Hosseini et al. 2021)**.** Alqhatani et al. (2023) reported that CS NPs coated with CU could reduce fenpropathrin-induced hepatotoxicity in rats via controlling lipogenesis and pyroptosis**.** CS NPs loaded with CU demonstrated remarkable protective effects against cypermethrin-induced oxidative stress damage in kidneys of rabbits (Anwar et al. 2020)**.**

## Conclusion

The present study provides a novel nanoformulation for curcumin using CS NPs encapsulation that selectively targets pathways on injured cells and enhances antioxidant activity and regulation of inflammatory and apoptotic markers. The encapsulation of CU with CSNPs significantly increased the mitigating effect of CU against Ni-induced hepatorenal toxicity. CS/CU NCs had a metal chelating potential as they reduced the Ni levels in hepatorenal tissue. More hepatic and renal disease models are required to justify the beneficial effect of CS/CU NCs and explore another mechanistic way. Further studies should be conducted to determine the protective effect of lower doses of CS/CU NCs to minimize the NPs exposure risk.

## Data Availability

All source data for this work (or generated in this study) are available upon reasonable request.
